# Complementary transcriptomic and proteomic analyses reveal the cellular and molecular processes that drive growth and development of *Fasciola hepatica* in the host liver

**DOI:** 10.1186/s12864-020-07326-y

**Published:** 2021-01-11

**Authors:** Krystyna Cwiklinski, Mark W. Robinson, Sheila Donnelly, John P. Dalton

**Affiliations:** 1 Zoology Department, School of Natural Sciences, Centre for One Health, Ryan Institute, National University of Ireland Galway, Galway, Ireland; 2 School of Biological Sciences, Queen’s University Belfast, Belfast, Northern Ireland, UK; 3 The School of Life Sciences, University of Technology, Sydney, Australia

**Keywords:** *Fasciola hepatica*, *Fasciola gigantica*, Trematodes, Transcriptomics, Proteomics, Liver, Growth, Development, Neoblasts

## Abstract

**Background:**

The major pathogenesis associated with *Fasciola hepatica* infection results from the extensive tissue damage caused by the tunnelling and feeding activity of immature flukes during their migration, growth and development in the liver. This is compounded by the pathology caused by host innate and adaptive immune responses that struggle to simultaneously counter infection and repair tissue damage.

**Results:**

Complementary transcriptomic and proteomic approaches defined the *F. hepatica* factors associated with their migration in the liver, and the resulting immune-pathogenesis. Immature liver-stage flukes express ~ 8000 transcripts that are enriched for transcription and translation processes reflective of intensive protein production and signal transduction pathways. Key pathways that regulate neoblast/pluripotent cells, including the PI3K-Akt signalling pathway, are particularly dominant and emphasise the importance of neoblast-like cells for the parasite’s rapid development. The liver-stage parasites display different secretome profiles, reflecting their distinct niche within the host, and supports the view that cathepsin peptidases, cathepsin peptidase inhibitors, saposins and leucine aminopeptidases play a central role in the parasite’s destructive migration, and digestion of host tissue and blood. Immature flukes are also primed for countering immune attack by secreting immunomodulating fatty acid binding proteins (FABP) and helminth defence molecules (FhHDM). Combined with published host microarray data, our results suggest that considerable immune cell infiltration and subsequent fibrosis of the liver tissue exacerbates oxidative stress within parenchyma that compels the expression of a range of antioxidant molecules within both host and parasite.

**Conclusions:**

The migration of immature *F. hepatica* parasites within the liver is associated with an increase in protein production, expression of signalling pathways and neoblast proliferation that drive their rapid growth and development. The secretion of a defined set of molecules, particularly cathepsin L peptidases, peptidase-inhibitors, saponins, immune-regulators and antioxidants allow the parasite to negotiate the liver micro-environment, immune attack and increasing levels of oxidative stress. This data contributes to the growing *F. hepatica* -omics information that can be exploited to understand parasite development more fully and for the design of novel control strategies to prevent host liver tissue destruction and pathology.

## Background

Helminth parasites of the genus *Fasciola* are the causative agents of fasciolosis, an economically important disease of ruminants and a WHO-recognised neglected tropical zoonotic disease [1]. Infection of the mammalian host follows ingestion of vegetation contaminated with an encysted stage, the metacercariae, from which the newly excysted juveniles (NEJ) emerge and penetrate through the intestinal wall and migrate to the liver. Within the liver, the parasite’s growth advances rapidly, doubling in size approximately every 2 weeks, alongside the development of parasite digestive and reproductive structures [2]. To facilitate this rapid growth and development the parasite feeds on liver tissue and blood. The extensive tunnelling activity results in severe haemorrhaging, as well as a marked immune cell infiltrate, comprised of lymphocytes, macrophages and particularly high levels of eosinophils [3], which eventually leads to visible fibrotic hepatic tracts.

The clinical manifestations associated with the acute phase of fasciolosis includes ill thrift and anaemia, and in some cases the excessive damage resulting from high parasite burdens leads to death in young lambs [3, 4]. In humans, typical symptoms associated with the intense internal bleeding of the liver include fever, nausea, extreme abdominal pain, hepatomegaly and skin rashes [5, 6]. To date the only effective anthelmintic for reducing the damaging clinical signs associated with the early stages of fasciolosis in animals and humans is triclabendazole which kills parasites from 2 to 3 weeks post-infection onwards [7]. The global spread of triclabendazole resistance [8], however, means that new methods of controlling fasciolosis in livestock and for the treatment of drug-resistant human fasciolosis are urgently needed.

Our knowledge of *F. hepatica* biology has been greatly advanced through the availability of extensive genome, transcriptome and proteome data [9, 10]. Analysis of these has provided detailed new insights into the virulence, growth and development of this parasite in the mammalian host. Our studies of the infective stages, namely the metacercariae and NEJ, have revealed that the parasite is transcriptionally active prior to infection and is primed for tissue penetration and migration through the host intestinal wall [10]. However, due to the importance of immature *F. hepatica* in the clinical manifestations and pathology of fasciolosis, we have focused this study on analysing previously published and new transcriptomic and proteomic data from both *F. hepatica* and *Fasciola gigantica* to elucidate the key processes critical for the growth and development of the parasite in the liver. We found that this life-cycle stage is particularly transcriptionally active with a significant enrichment of metabolic pathways associated with protein production, signal transduction and neoblast proliferation. Complementary proteomic analyses of the secretome identified a distinct profile of secreted proteins that support the immature fluke’s capacity for tissue penetration, blood feeding and regulation of the host immune responses. We also probed previously published microarray data generated from liver tissue of infected animals [11], and have correlated the damage caused by the migrating parasites with key host and parasite antioxidant molecules that attenuate the oxidative stress associated with fasciolosis. These new results and insights into liver migration by *F. hepatica* can be exploited for the development of treatments that aim to prevent the pathogenesis associated with fasciolosis in animals and humans.

## Results and discussion

### Immature flukes are highly transcriptionally active

**Fig. 1:**
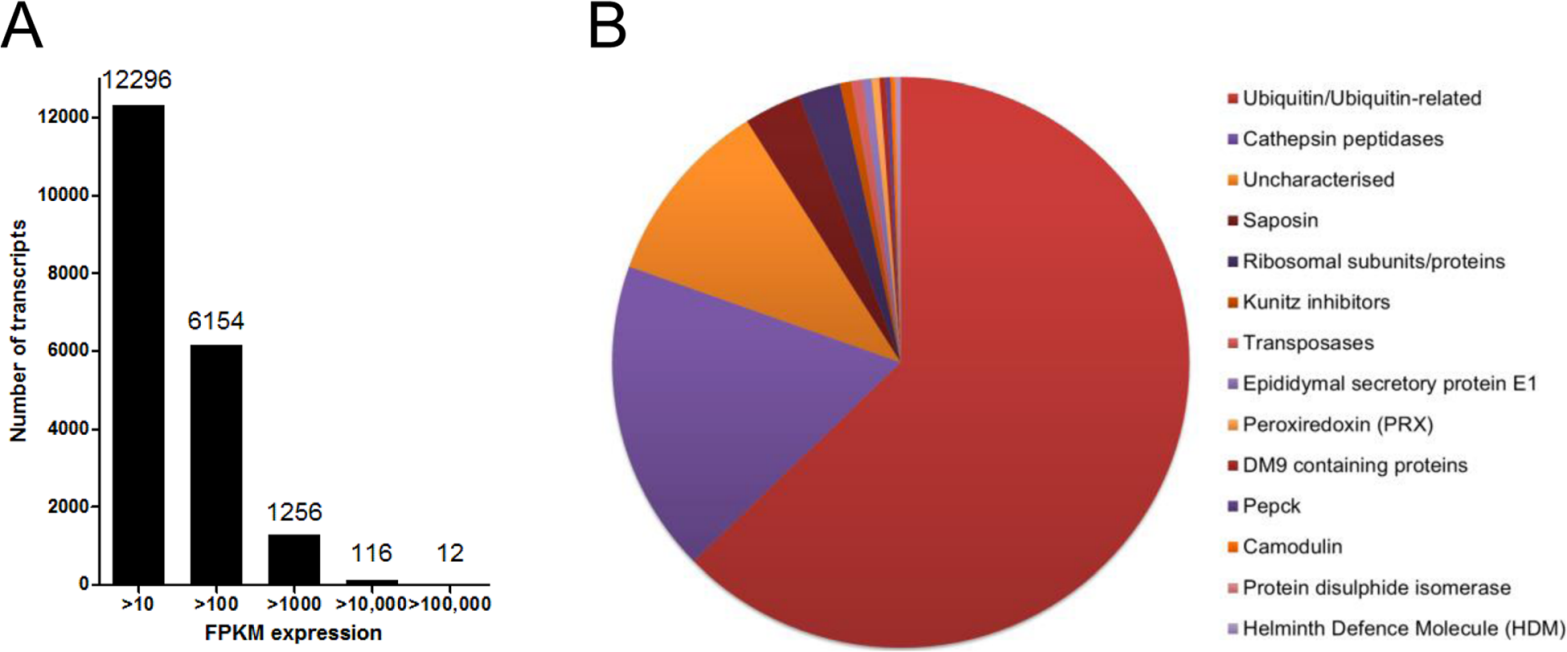
*Fasciola hepatica* immature parasites are transcriptionally active. **a** Graphical representation of the number of transcripts expressed by the immature parasite stages (average of three biological replicates) by FPKM values. **b** Schematic detailing the profile of the top 100 transcripts based on the average FPKM values for three biological replicates, corresponding to 59% of the total gene transcription of the immature parasites

To investigate the molecular mechanisms related to migration in the liver by immature stage *F. hepatica,* we carried out transcriptome analysis by RNASeq of parasites recovered from the livers of mice 21-days post-infection. An average of 41.7 million high quality reads were generated for each biological replicate of immature *F. hepatica* parasites, that were mapped to the annotated gene models identified in the draft *F. hepatica* genome (v1; PRJEB6687). A subset of 27,407 transcripts were used for further analysis based on a transcription of greater than 1 FPKM in at least two of the biological replicates (Additional file 2). Consistent with our previous analysis of the *F. hepatica* life cycle stages [9], we observed that the immature flukes are particularly transcriptionally active, with over 7500 transcripts exhibiting a value > 100 FPKM (Fig. 1).

Analysis of the putative function of the 27,407 transcripts, highlighted a significant enrichment in gene ontology (GO) terms related to binding, metabolic process and catalytic activity. In particular, key GO terms associated with transcription (GO:0006355, regulation of transcription; GO:0003677, DNA binding; GO:0003676, nucleic acid binding), translation (GO:0006412, translation; GO:0005840, ribosome; GO:0003735, structural constituent of ribosome), proteolysis (GO:0006508), lipid metabolic processes (GO:0006629) and signal transduction (GO:0007165) were amongst the most enriched (*P* < 0.05, FDR adjusted) (Additional files 3 and 4). The enrichment of genes related to transcription and translation is consistent with the parasite increasing the number of genes it transcribes in comparison to the earlier invasive NEJ stage and reflects its intense growth and development in the liver. Ubiquitin predominates amongst the most abundant 100 transcripts, which represent 59% of the total transcription of the immature flukes (Fig. 1). It plays a key role in regulating proteins at the cellular level via the ubiquitin proteasome system and is specifically important for controlling cell cycle progression during intensified cell growth and proliferation [12, 13].

Protein metabolism is a highly energy-dependent process and since parasitic trematodes are unable to synthesise lipids, specifically long chain fatty acids and cholesterol that they use as an essential energy source [14], these must be acquired directly from the host. The abundance of genes associated with lipid metabolic processes, therefore, emphasises that the immature flukes have transitioned from relying on endogenous energy sources to a dependence on the host for nutrients. This is in agreement with earlier ultrastructural observations that showed that the gastrodermal cells of immature *F. hepatica* only begin to cycle between secretory and absorptive phases (required for uptake of host-derived nutrients) after 2 weeks development in the murine host [15].

Several highly-transcribed genes were also identified that are typically found within the *F. hepatica* excreted-secreted proteins (ES) or secretome and act at the host-parasite interface (Fig. 1, see below). These included cathepsin peptidases (cathepsin L2, FhCL2, being the most highly transcribed), saposins, Kunitz-type inhibitor of the FhKT1 group FhKT1.2, peroxiredoxin (FhPRX), the helminth defence molecule (FhHDM) and calmodulin (FhCaM3). These proteins play a role in facilitating blood feeding, heme scavenging and regulating the host immune response by the parasite.

Calmodulins have also been linked to the growth and development of several helminths [16–18]. RNAi experiments in *F. hepatica* NEJ suggests a role in the growth and motility of the parasite [18] while in adult worms, FhCaM3 may play a role in calcium signalling during egg formation since they have been located within the eggs and vitelline cells [19]. However, their role in immature liver stage flukes is currently unknown, although FhCaM2 and FhCaM3 proteins have been shown to be constitutively expressed at this stage [18].

**Fig. 2:**
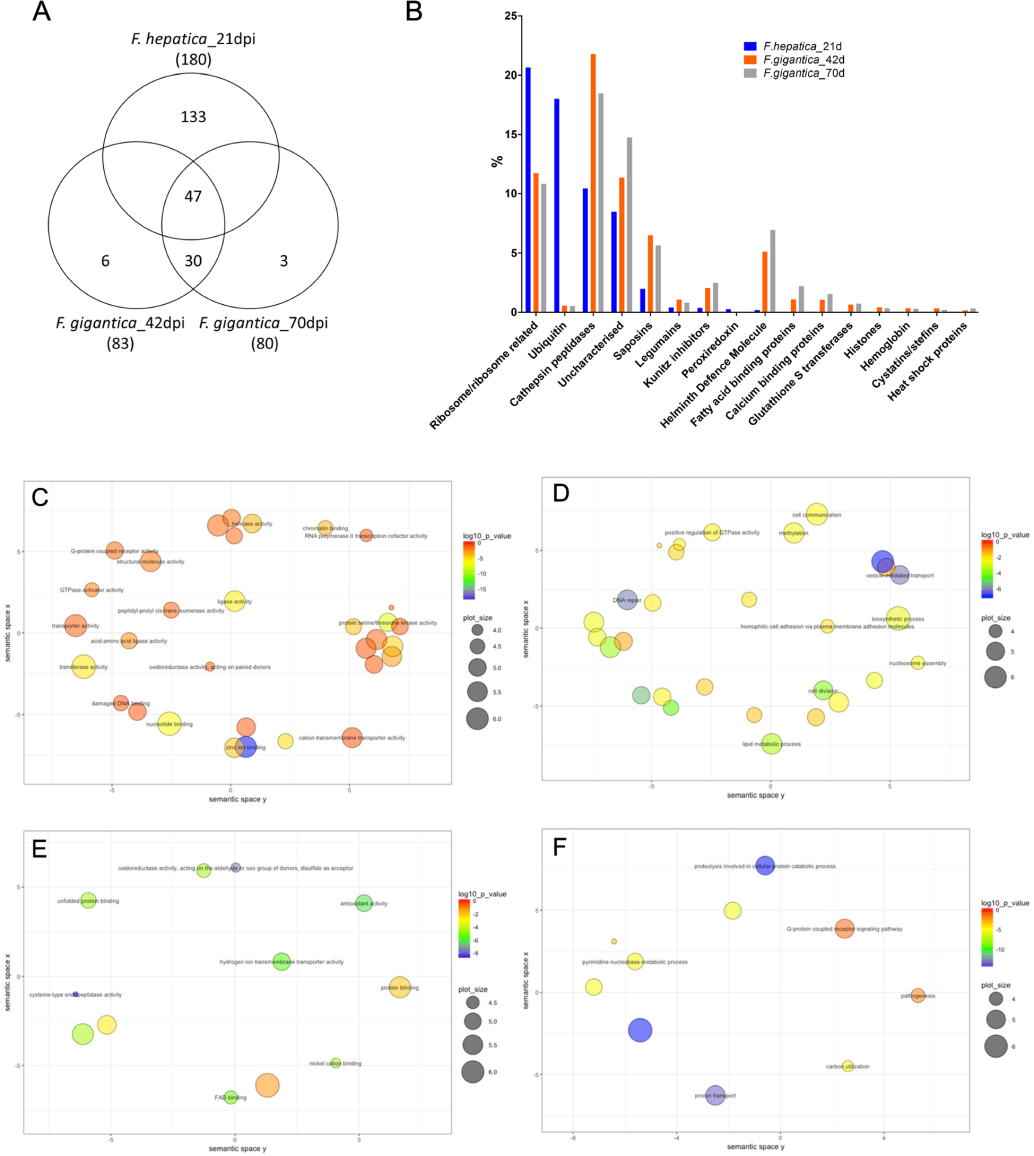
The immature *F. hepatica* parasites display a different profile of gene expression compared with *F. gigantica*. **a** Venn diagram representing the number of significantly enriched GO terms shared between the *F. hepatica* immature flukes at 21 days post infection (*F. hepatica*_21dpi) and the *F. gigantica* immature flukes at 42- and 70-days post infection (*F. gigantica*_42dpi; *F. gigantica*_70dpi). The numbers in brackets depict the total number of enriched GO terms per dataset. Description of the GO terms is presented in Additional file 4. **b** Graphical representation of the top 150 abundantly transcribed genes from *F. hepatica* immature flukes at 21 days post infection (*F. hepatica*_21dpi) and the *F. gigantica* immature flukes at 42- and 70-days post infection (*F. gigantica*_42dpi; *F. gigantica*_70dpi). Data is represented as the percentage abundance relative to total gene transcription for each dataset, with genes grouped by gene family where possible. **c-f** Schematic representation of the gene ontology (GO) enrichment analysis using REVIGO based on molecular function and biological processes highlighting the enriched GO terms that play a role as the parasite grows and develops. **c** Molecular function GO terms within the *F. hepatica* immature transcriptome. **d** Biological process GO terms within the *F. hepatica* immature fluke transcriptome. **e** Molecular function GO terms within the *F. gigantica* immature fluke transcriptomes. **f** Biological process GO terms within the *F. gigantica* immature transcriptomes. The bubble colour indicates the log value of the FDR adjusted *p* value and the circle size (plot size) represents the frequency of the GO term within the gene ontology annotation database (GOA; more general terms represented by larger plot size)

To further elucidate the key biological processes and molecular functions critical for the liver migrating immature flukes, we carried out a comparative analysis with transcriptome data from the *F. gigantica* immature (liver-stage) flukes recovered from buffalo at 42- and 70-days post infection [20] (Fig. 2). Since *F. hepatica* was sourced from mice at 21 days after experimental infection and *F. gigantica* from buffalo at 42 and 70 days after natural infection the observed transcriptional differences may be host, or age related; as such we carried out a broad analysis based on GO enrichment and the most abundantly transcribed genes to allow a relative comparison between the datasets. A total of 47 GO terms were similarly enriched within the *F. hepatica* and *F. gigantica* datasets. Significant enrichment associated with translation (GO:0006412), proteolysis (GO:0006508) and signal transduction (GO:0007165), and molecular functions such as calcium ion binding (GO:0005509), catalytic activity (GO:0003824) and cysteine-type peptidase activity (GO:0008234) was observed (Fig. 2; Additional file 4), highlighting the central roles that these processes/functions play in the liver migrating stages of both species. We found metal ion binding, specifically zinc ion binding (GO:0008270), and vesicle-mediated transport (GO:0016192) were enriched within *F. hepatica*, whereas distinct enrichment of proteolysis involved in cellular protein catabolic process (GO:0051603), oxidation-reduction process (GO:0051603) and protein transport (GO:0015992) was observed in *F. gigantica.*

Comparative analysis of the most abundantly transcribed 150 transcripts from the *F. hepatica* and *F. gigantica* datasets (Fig. 2) revealed that ribosome-associated genes, cathepsin peptidases and saposins play an important role for the immature flukes of both species. Consistent with the analysis of the *F. hepatica* immature transcriptome, the peptidase inhibitors, specifically the Kunitz-type inhibitors and cystatins/stefins, are also highly transcribed within the *F. gigantica* immature flukes at 42 and 70 dpi (with relatively higher levels of transcription observed during these later stages). Similarly, the helminth defence molecule (HDM) is highly transcribed by the *F. gigantica* 42 and 70 dpi stages. However, in contrast to *F. hepatica*, immature *F. gigantica* displayed lower levels of transcription of the ubiquitin-associated genes.

Transcription of redox-based antioxidants shows that immature *F. hepatica* favour the thioredoxin-dependent antioxidant defence system involving thioredoxin and peroxiredoxin, whereas, *F. gigantica* is more dependent on glutathione as glutathione S transferases (GST) are more highly transcribed.

### Key metabolic pathways associated with growth & development

**Fig. 3:**
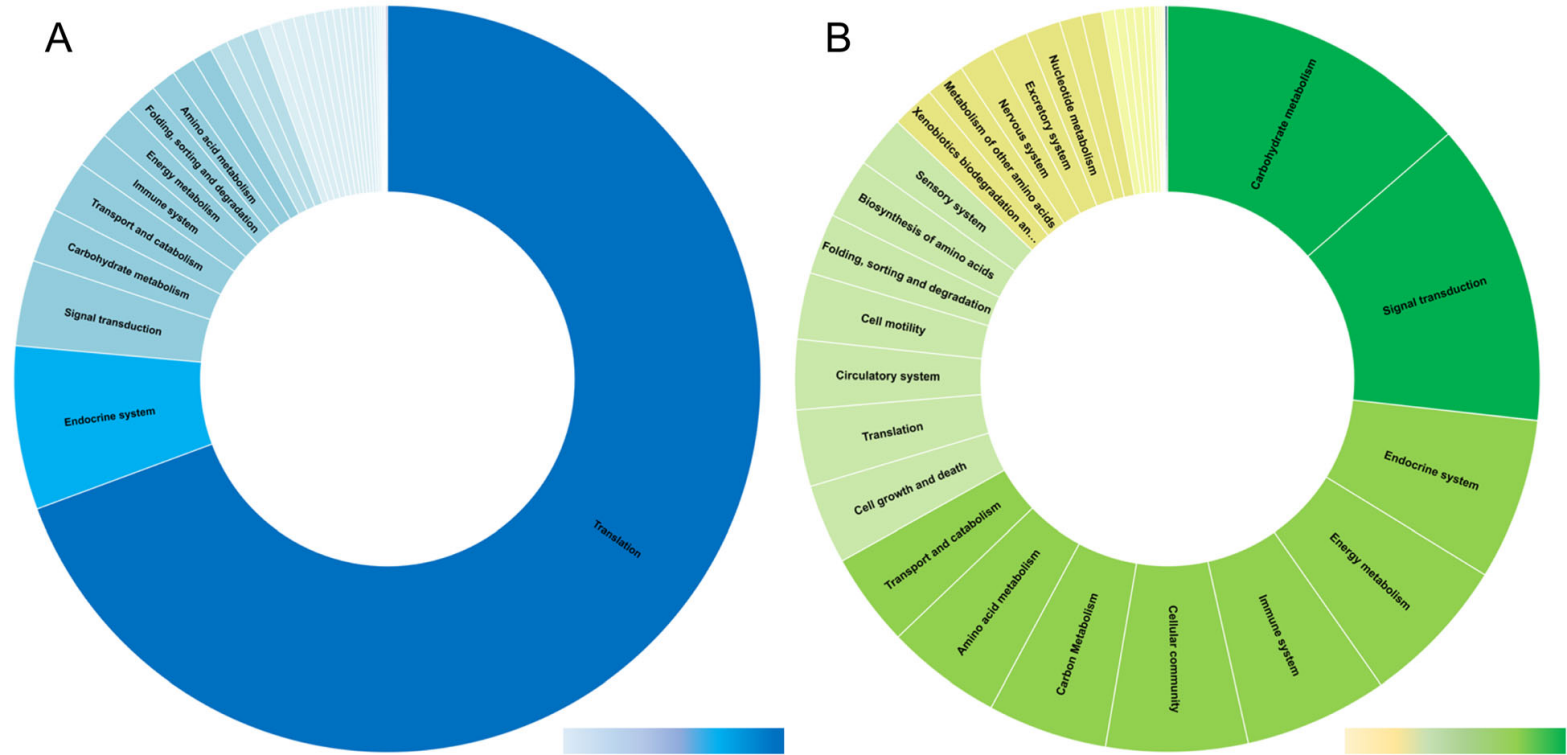
An abundance of transcripts and proteins are associated with metabolism within the immature transcriptome and somatic proteome. **a** Schematic representation of the transcription of genes associated with metabolism (KEGG module, ko00001), normalised at the KEGG module level relative to the total metabolic transcription. Relative expression is shown by light blue to dark blue depicting low to high levels of transcription, respectively. **b** Schematic representation of the somatic protein abundance (based on emPAI values) corresponding to the proteins associated with metabolism (KEGG module, ko00001), normalised at the KEGG module level relative to the total protein abundance associated with metabolism. Relative protein abundance is shown by yellow to dark green, depicting low to high protein abundance, respectively

To gain insight into the critical metabolic pathways associated with liver migration, we analysed the KEGG metabolic pathways that were highly represented within the immature fluke transcriptome and somatic proteome (Fig. 3; Additional files 2 and 5). Consistent with the gene ontology data, the translation pathways (ko09122) are the most highly transcribed, specifically genes associated with the ribosome (ko03010), further emphasising the rapid protein production the parasite undertakes. High levels of transcription were also observed for pathways that are associated with the endocrine system (ko09152) and signal transduction (ko09132) that regulate lipid metabolism and cellular proliferation, predominated by the genes associated with the PPAR signalling pathway (ko03320) and PI3K-Akt signalling pathway (ko04151), respectively.

The increased transcription of these metabolic pathways correlates with their protein expression within the somatic proteome with carbohydrate metabolism (ko09101) and signal transduction (ko09132) amongst the most highly expressed based on emPAI values. Contributing to carbohydrate metabolism are proteins involved in Glycolysis (ko00010), TCA cycle (ko00020), and the Glyoxylate and Dicarboxylate metabolism (ko00630) pathways. Early studies by Tielens et al. [21] have shown that as *F. hepatica* grows and develops, the processes used for energy metabolism switch from aerobic to anaerobic dismutation. Aerobic acetate production predominates during the immature fluke stage, with the parasite utilising acetate as its primary carbon source. The identification of proteins associated with both the TCA cycle and the Glyoxylate and Dicarboxylate metabolism pathway reflects this transitioning phase; both pathways involve the conversion of isocitrate to malate, though the glyoxylate cycle occurs under anaerobic conditions in contrast to the aerobic process of the TCA cycle [22].

**Fig. 4:**
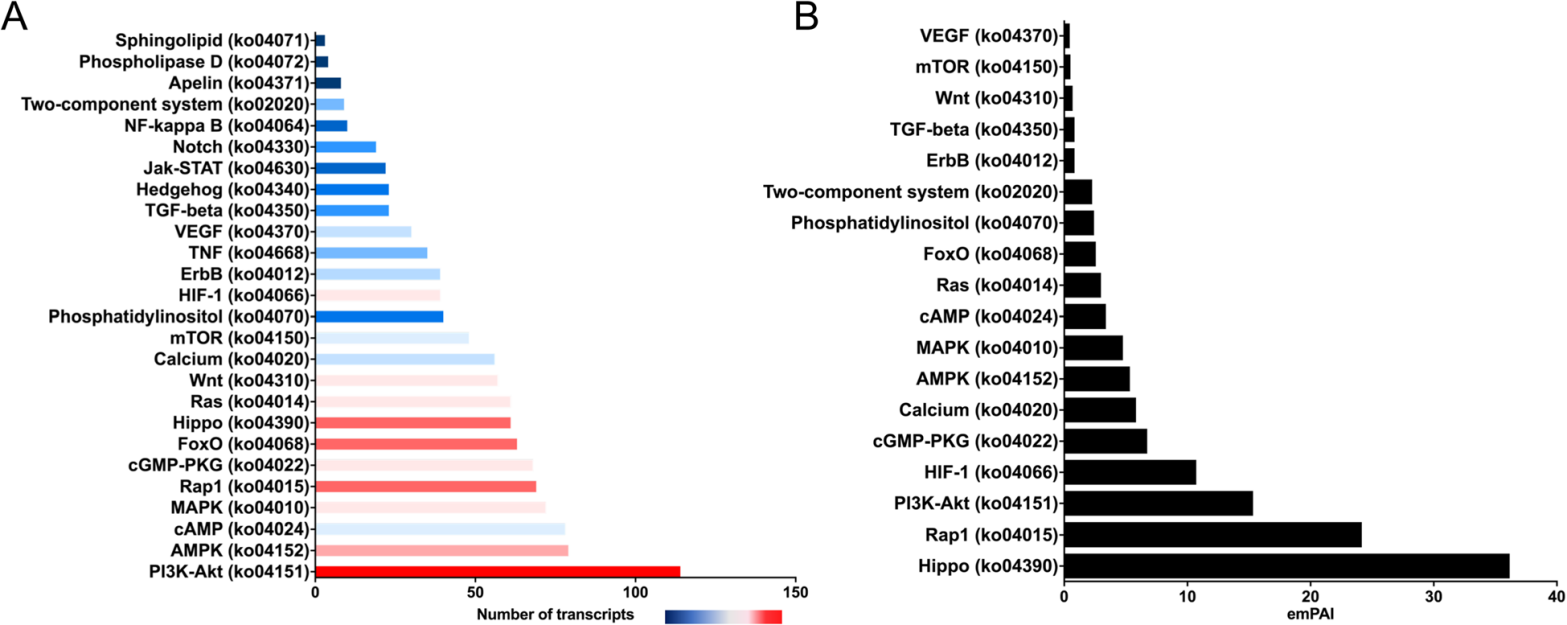
Signal transduction pathways are significantly enriched in immature liver-stage *F. hepatica* (**a**) Graphical representation of the number of transcripts associated with the signal transduction pathways as per the KEGG pathway codes, highlighted by their relative FPKM expression, shown by a blue to red scale depicting low to high levels of expression, respectively. **b** Graphical representation of the protein abundance of the signal transduction pathways as per the KEGG pathway codes, displayed as emPAI values from the proteomic analysis

The transcriptomic enrichment of signal transduction pathways that regulate cellular differentiation and proliferation that mediate growth, development and metabolism [23] correlates with our somatic proteome data (Fig. 4). In particular, the PI3K-Akt signalling pathway (Fig. 4a), represented by the largest number of signal transduction associated-transcripts, is amongst the most abundant signal transduction pathway within the somatic proteome (Fig. 4b). This pathway plays an important role in regulating neoblast/pluripotent cells in the planarian *Schmidtea mediterranea* [24] and is essential for potentiating the survival of these pluripotent cells [25]. The generation and proliferation of neoblast/pluripotent cells by *F. hepatica* is observed throughout its life cycle [2, 10] and, therefore, the neoblast-regulating PI3K-Akt signalling pathway, together with the upregulation of key genes associated with neoblast proliferation [10], support the idea that these play a crucial role for the growth and development of the immature flukes.

Other key signal transduction pathways critical for growth and development that are enhanced in the immature flukes include the (a) AMPK signalling pathway that regulates energy homeostasis and metabolism [26, 27] and plays a critical role in the regulation of cell growth [28]. Recently, Kadekar and Roy [29] have shown that this pathway is also involved in regulating germline stem cells in *Caenorhabditis elegans* within the energy-stressed dauer stage via the small RNA pathway; (b) Hippo signalling pathway that regulates organ size through regulation of cellular proliferation and expansion of neoblasts/pluripotent cells during stages of development [30–33]. As the immature parasites migrate through the liver some of the reproductive organs are at an advanced stage of development, notably the testes which show a clear follicular appearance by 21 dpi in mice [34]. This pathway could regulate reproductive development that must be finely tuned to ensure rapid egg production upon the arrival of the flukes in the bile duct. Hippo signalling may also control the size of the parasite relative to its host, especially considering that *F. hepatica* can infect a range of mammalian hosts; and (c) HIF-1 signalling pathway that is induced under decreased oxygen partial pressures, and is responsible for regulating oxygen-regulated metabolic gene expression [23]. This pathway may be important as the parasite increases in size, which decreases the parasite surface to volume ratio and thereby limits the diffusion of oxygen to the internal tissues and organs of the parasite [21].

### Immature flukes are primed for blood feeding, tissue degradation and immune evasion

**Fig. 5:**
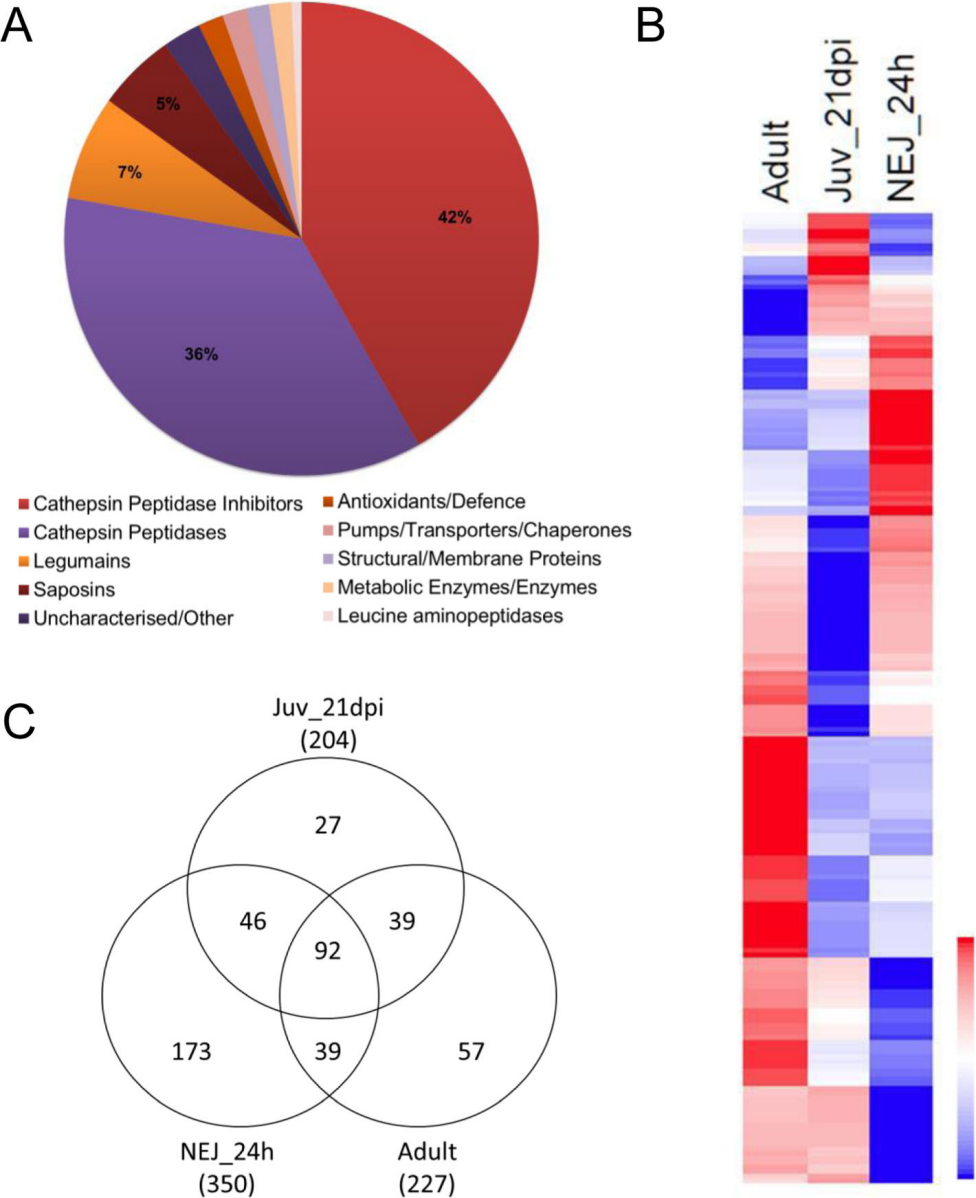
The *F. hepatica* life cycle stages have distinct secretome profiles. **a** Graphical representation of the top 50 proteins secreted by the immature flukes representing 87% of the total protein secreted (based on emPAI values of three biological replicates), highlight that the immature fluke secretome is predominantly comprised of cathepsin peptidases and cathepsin peptidase inhibitors. **b** Graphical representation of the composition of the *F. hepatica* stage-specific secretomes, based on the emPAI abundance. Relative abundance is shown by a blue to red scale, depicting low to high levels of abundance, respectively. **c** Venn diagram representing the comparative analyses of proteins secreted by newly excysted juveniles 24 h post excystment (NEJ 24 h), the immature parasites (Juv21dpi) and the adult parasites. The protein count is based on a cut-off of at least 2 unique peptides

To extend our earlier gel-based studies of the immature fluke secretome that identified 45 proteins [35], we carried out an in-depth gel-free proteomic analysis. This approach resulted in the identification of a total of 210 proteins, based on the acceptance criteria of two unique peptides within at least two biological replicates, with the top 50 proteins representing 87% of the total protein secreted (protein abundance, emPAI; Additional file 6). Functional analysis of these most abundant proteins reveals that they are mostly comprised of cathepsin peptidases and cathepsin peptidase inhibitors, representing 36 and 42% of the total protein, respectively (Fig. 5a).

As we have reported previously that, in contrast to other trematodes, *F. hepatica* relies almost exclusively on cathepsin cysteine peptidases for tissue degradation, migration and feeding within the mammalian host [36, 37]. The higher levels of these enzymes secreted by the immature parasites further highlights their importance in the tissue degradation process. The most abundant cathepsin peptidases identified were two members of the cathepsin L3 group (Nomenclature as per [36]; FhCL3_4, BN1106_s3008B000074/ BN1106_s4187B000060) and a single cathepsin L2 (FhCL2; BN1106_s8098B000020); this is not surprising since these two peptidase groups possess unique and potent collagenolytic activity that allows the parasite to effectively degrade insoluble collagen within the liver extracellular matrix and disintegrate the tissue structure [38]. While FhCL1 was also identified within the immature fluke secretome this was present at lower protein levels compared with FhCL2 and FhCL3 (2 fold less and 7.5 fold less, respectively). We have shown that the substrate specificity of FhCL1 is adapted to digest host haemoglobin to peptides and is thus expressed most abundantly by the obligate blood-feeding adult fluke [39]. However, the suite of FhCL1/2/3 peptidases would confer the immature fluke with a very effective means of tissue and blood feeding, and this is further complemented by several saposins and leucine aminopeptidases that are important for the lysis of blood cells and the terminal hydrolysis of haemoglobin-derived peptides, respectively [40–42].

The application of cathepsin peptidases in a variety of functions requires strict regulation to ensure that excessive damage to both parasite and host tissues does not occur. Cathepsin L peptidases are produced as inactive zymogens that are autocatalytically activated within the low-pH gut of the parasite to mature enzymes prior to their release by regurgitation [37, 39]. *F. hepatica* controls the hydrolytic activity of these peptidases by co-secreting of a range of peptidase inhibitors, specifically cystatins/stefins and Kunitz-type inhibitors. Here we discovered that the most abundant of these in the immature secretome is a member of the Kunitz-type protease inhibitor family, specifically FhKT1 group member FhKT1.2 (BN1106_s318B000274), which represents 33% of the total secreted protein. We have previously shown that, unlike other Kunitz-type protease inhibitors that typically inhibit serine proteases, the FhKT1 group are specifically adapted to inhibit cathepsin L peptidases which further highlights the importance of a peptide/anti-peptidase mechanism regulating parasite-mediated tissue degradation [43]. The abundance of secreted FhKT1.2 may also indicate a pivotal role in the control of host immune response polarisation as Cervi and colleagues [44] showed that this inhibitor can elicit a regulatory IL-27-dependent phenotype in LPS-stimulated dendritic cells which prevented the development of Th1 and Th17 responses. This could complement the ability of cathepsin L peptidases to prevent MyD88-independent TRIF-dependent signalling pathways of Toll-like receptor (TLR) 3 and 4 in macrophages, which also impairs the development of Th1 responses in mice [45].

The cathepsin L peptidases also play a role in the modulation of the host immune response by cleaving immunoglobulins at their hinge region and thus disconnecting the antibody binding Fab domain from the Fc domain that is essential for attracting innate phagocytes [46]. Other key molecules within the top 50 secreted proteins that likely play a role in the modulation/suppression of the host immune response are the helminth defence molecule (FhHDM) and several fatty acid binding proteins (FABP; Fh2, Fh3, Fh15). FhHDM binds to the cell surface and enters the endo-lysosomal system of macrophages where it inactivates lysosome proteases to selectively reduce pro-inflammatory responses [47, 48]. FhHDM also binds iron and thus has been suggested to function in the scavenging and transportation of heme [49]. As FhHDM is elevated within the NEJ secretome, and can neutralise the inflammatory effects of bacterial lipopolysaccharide, we have postulated a role in preventing potential endotoxin-induced inflammatory responses from bacteria that may translocate from the intestinal lumen to the peritoneal cavity with the migrating parasite [10, 50, 51]. The role of FhHDM during the liver migratory stage has not been specifically investigated although the parasite is likely to benefit from its broader suppressive effects on the activation of host macrophages as it migrates through the tissue.

Espino and colleagues [52–55] have shown that the FABP also play a role in reducing LPS-induced inflammation by suppressing Toll-like receptor (TLR) stimulation in macrophages and inhibiting the inflammatory cytokines TNF and IL-1β. Acting as an antagonist of TLR4, both native and recombinant FABP suppresses the TLR4 signalling cascade by blocking the interaction of LPS with the CD14 co-receptor. However, proteomic analysis of the FABP superfamily reveals that the adult parasites secrete these molecules in greater abundance in comparison to the NEJ, which is also highlighted by our secretome analysis (Additional file 6), indicating a possible blood feeding role in the uptake of fatty acids from host blood rather than an immunomodulatory role when the parasite is in the bile duct [56].

### Distinct profile of secreted proteins reflects liver fluke stage-specific niches/roles

The major life cycle stages of *F. hepatica*, namely the NEJ, immature flukes and adult flukes occupy very different niches within the host. This is reflected in their secretomes and while comparative analysis of the three stages revealed 92 shared proteins, each stage also secreted a distinct profile of proteins (Fig. 5; Additional file 6). However, the immature flukes secrete the lowest amount of distinct proteins compared with both NEJ and adult. The 27 proteins found to be exclusively secreted by the immature flukes includes a range of uncharacterised proteins, a specific member of the cathepsin L3 peptidase group (FhCL3_2; BN1106_s8881B000009), FhKT1 group member FhKT1.1 (BN1106_s8826B000029), a saposin (BN1106_s4986B000028) and a ferritin protein (BN1106_s3950B000041). The NEJ-specific proteins also include a range of uncharacterised proteins, in addition to a serpin (FhSrp3, based on nomenclature of [57], a member of the cathepsin L3 peptidases (FhCL3_3; BN1106_s10139B000014) and thioredoxin glutathione reductase (TGR). Key proteins exclusively secreted in high amounts by the adults include a ferritin (BN1106_s709B000627) and a mu class GST (BN1106_s4370B000168).

For all three life cycle stages, the cathepsin peptidases are amongst the most abundant proteins secreted, consistent with other studies [10, 35, 58]. However, the type of cathepsin peptidases secreted reflects the specific niche of each life cycle stage, with the NEJ secreting predominantly the collagenolytic cathepsin L3 and three cathepsin B peptidases (FhCB1, FhCB2 and FhCB3), and the adult parasites abundantly secreting the critical peptidases involved in blood feeding, namely cathepsin L1, and L5 peptidases, and collagenolytic cathepsin L2 peptidase. Interestingly, the profile of the immature flukes reflects the transitional state between NEJ and adult, with a range of both the NEJ-associated peptidases such as cathepsin L3 and cathepsin B2 being secreted in addition to the adult fluke-associated cathepsin L1, L2 and L5 peptidases. This wider collection of peptidases undoubtedly provides the immature fluke with a more potent proteolytic armoury during what is arguably the most challenging time in its intra-mammalian development.

A key feature of the secreted proteins of *F. hepatica* are peptidase inhibitors, which can act upon both host and parasite proteases. Three types of protease inhibitors are secreted in abundance by each *F. hepatica* life cycle stage, namely the stefins/cystatins (cathepsin peptidase inhibitors), the atypical Kunitz-type cathepsin inhibitors and the serine protease inhibitors (serpins). All three of these protease inhibitors are encoded by multi-copy gene families, members of which display stage-specific expression. Although each of the life cycle stages secretes members of all three groups of inhibitors, the amounts vary with each developmental stage (Additional file 7). The NEJ and adults secrete comparable levels of stefins/cystatins, which is comprised predominately of cathepsin L peptidase inhibitor, FhStefin-1. Similarly, both these stages also secrete members of the serpin family, which have distinct inhibitory profiles, specifically being potent inhibitors of host chymotrypsin and kallikrein [57, 59]. FhSrp1 and FhSrp3 are secreted in greatest abundance by the NEJ compared with the FhSrp4 by the adults. In contrast, the immature flukes secrete these inhibitors in relatively low amounts, relying more on the Kunitz-type cathepsin peptidase inhibitors. The abundance of the *F. hepatica* Kunitz-type inhibitors that can inhibit cathepsin L peptidases of both host and parasite implies that these cathepsin peptidases play a crucial role within the liver tissues.

### Proline synthesis, oxidative stress and bile duct hyperplasia

**Fig. 6:**
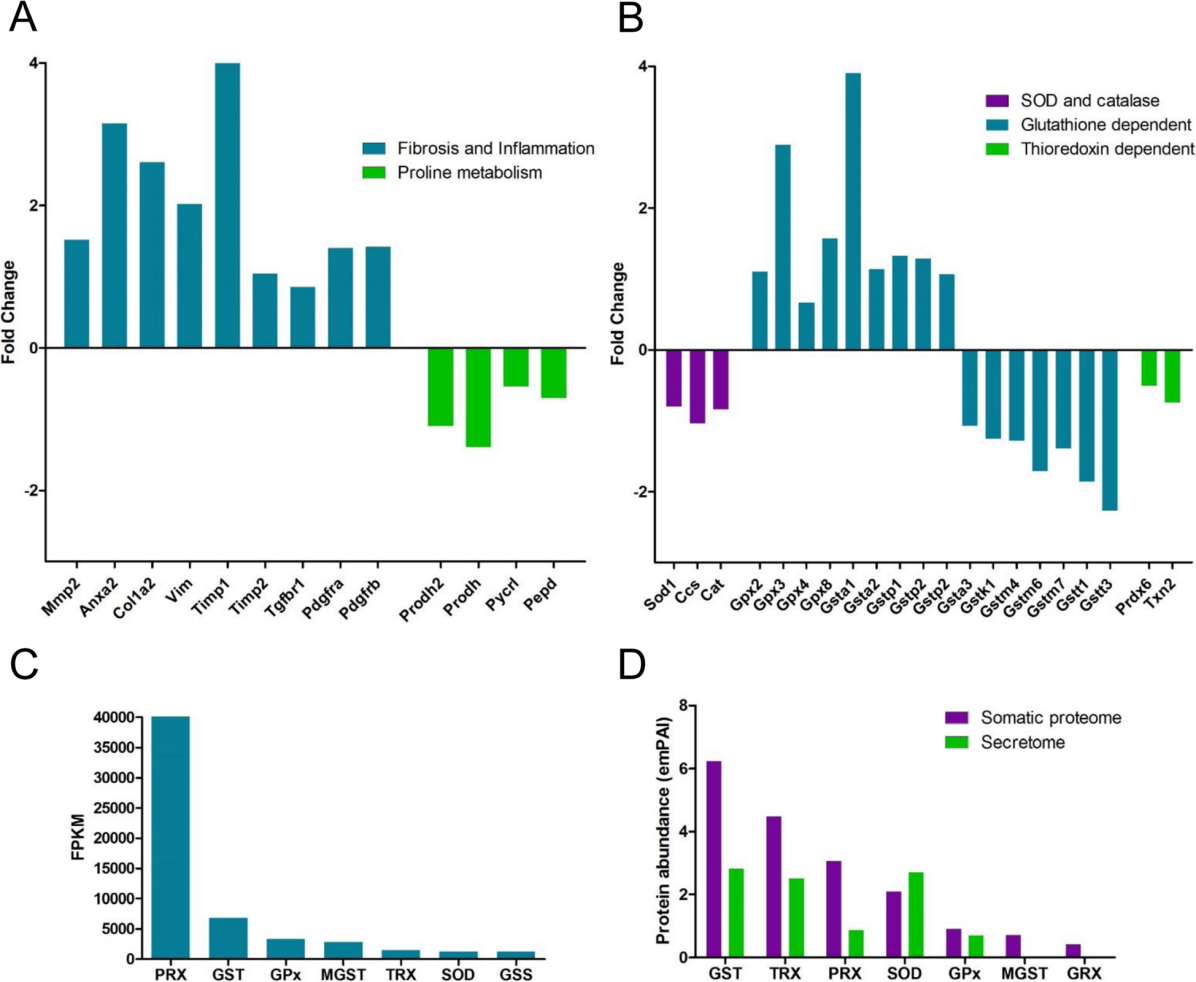
Host and parasite genes that play a critical role for tolerating the oxidative stress induced by *F. hepatica* infection. **a** Graphical representation of the fold change in transcription of genes associated with fibrosis and inflammation, and proline metabolism within liver tissue from non-infected and *F. hepatica*-infected mice (**b**) Graphical representation of the fold change in transcription of genes associated with oxidative stress within liver tissue from non-infected and *F. hepatica*-infected mice. Data for **a** & **b** is extrapolated from the microarray data generated by Rojas-Caraballo and colleagues, [11]. Details of the gene abbreviations are provided in Additional file 8. **c** Graphical representation of the transcription of key antioxidant related genes within the *F. hepatica* immature fluke transcriptome, based on FPKM values. **d** Graphical representation of the protein abundance of key antioxidant related proteins within the *F. hepatica* immature fluke somatic proteome and secretome. Abbreviations for panels B and C: PRX, peroxiredoxin; GST, glutathione S transferases; GPx, glutathione peroxidase; MGST, microsomal glutathione S transferases; TRX, thioredoxin; SOD, superoxide dismutase; GSS, glutathione synthetase, GRX, glutaredoxin

Bile duct hyperplasia is a characteristic pathology associated with liver fluke infection and begins prior to the parasite entering the bile ducts. Several decades ago, studies by Isseroff and colleagues [60–63] reported that proline secreted by immature *F. hepatica*, as early as 25 dpi, stimulates collagen synthesis that results in the bile duct enlargement. Analysis of the immature fluke transcriptome data revealed that the arginine and proline metabolism pathway is amongst the most highly transcribed of the amino acid metabolism pathways. Transcription analysis of key enzymes within this pathway show that proline is synthesised from both glutamate and ornithine, with a predominance for the conversion from ornithine based on the FPKM transcription value and the presence of ornithine aminotransferase (EC 2.6.1.13; K00819) within the somatic proteome. In contrast, genes related to proline synthesis within the infected mouse liver, based on the liver tissue microarray data from *F. hepatica*-infected Balb/c mice (21 dpi) generated by Rojas-Carabello et al. [11], are down-regulated compared with the non-infected animals (Fig. 6a; Additional file 8). These data support Isseroff’s contention that the parasite is the source of proline rather than the host liver tissue.

The increased production of proline by the parasite may also be a response to environmental stress [64, 65]. In addition to generating reactive oxygen species (ROS), proline is also involved in stabilising antioxidant enzymes as well as the direct scavenging of ROS to maintain a balanced redox state [64, 65]. Studies of liver fibrosis using bile duct ligated rats have shown that proline supplements can alleviate early markers of oxidative stress and histopathogical changes associated with fibrosis [66]. Thus, in addition to enlargement of the bile duct, the proline secreted by the parasite may also be involved in mitigating oxidative stress caused by fibrosis.

### Acute stage of fasciolosis is associated with the expression of anti-oxidants by both host and parasite

Due to its high metabolic activity, the liver is highly susceptible to oxidative stress which is a well reported characteristic of many chronic liver diseases [67]. Oxidative stress is also a feature of fasciolosis as a result of the damage caused by the migrating parasites, the subsequent recruitment of immune cells to perforated areas and the induction of fibrogenesis to repair the damage [68–70]. We therefore interrogated the liver tissue microarray data from *F. hepatica*-infected Balb/c mice (21 dpi) generated by Rojas-Carabello et al. [11] to investigate the gene transcriptional profile associated with oxidative stress (Fig. 6b; Additional file 8). Consistent with their reported elevation of genes associated with liver toxicity, injury and death we also observed that genes associated with fibrosis and inflammation increased in infected mice (Fig. 6a; Additional file 8); these included key genes associated with hepatic stellate cells that are critical for the response to liver injury and collagen deposition (*ANXA2, VIM, COL1A2, TIMP1*). Fibrosis resulting from large numbers of parasites migrating through the liver (possibly from repeated liver fluke infections) therefore has the potential to exacerbate the level of oxidative stress in the tissue.

Antioxidants involved in the first line of defence against oxidative stress include superoxide dismutase (SOD), which catalyses free oxygen radicals to hydrogen peroxide, and catalase and glutathione peroxidase (GPx) that convert hydrogen peroxide to water. Analysis of the microarray data shows that *Sod*, the SOD-copper chaperone (*Ccs*), and catalase display lower levels of transcription in fluke-infected liver compared with the liver from non-infected animals (Fig. 6b). This correlates with the lower SOD protein activity measured in liver tissue and sera in *F. hepatica* and *F. gigantica*-infected cattle, rats and humans [71–74]. The reduced SOD-copper activity in the liver of fluke-infected animals could compromise their ability to deal with oxidative stress, particularly during co-infections with gastrointestinal parasites that have been shown to interfere with the absorption of copper and thereby reduce blood copper levels in sheep [75–77].

Genes encoding peroxiredoxin (PRX) and thioredoxin (TRX) also displayed lower levels of transcription in fluke-infected liver (Fig. 6b). By contrast, the genes encoding liver GPx and GSTs showed increased levels of transcription compared with the non-infected animals which may suggest a greater reliance on the glutathione thiol-dependent antioxidant system by the host during infection.

In response to the high oxidative stress-induced environment of the liver, the immature flukes express an antioxidant defence system that is comparable to that of its mammalian hosts (Fig. 6c & d). The components of the SOD and thiol-dependent antioxidant systems are expressed by the immature flukes, with peroxiredoxins representing the most highly transcribed antioxidants (Fig. 6c). Analysis of the somatic and secreted proteomes reveals that SOD and GPx are expressed at comparable levels within both datasets. Since, like other helminths, *Fasciola* species lack a catalase gene [9], hydrogen peroxide detoxification is performed by GPx in addition to the actions of the thioredoxin thiol-dependent antioxidant system [78]. Accordingly, and consistent with the transcriptome data, GSTs are the most abundant antioxidant protein family within the somatic proteome and are secreted at comparable levels to the TRX and SOD proteins. Contrary to its high transcription and presence within the somatic proteome, PRX is secreted at lower levels compared with TRX. However, the presence of the redox-based antioxidant proteins within the secreted proteins implies a protective/defensive role at the parasite surface that has the potential to regulate the surrounding host environment.

## Conclusions

Our analysis of the transcriptome and proteome (somatic and secretome) of the immature stage of *F. hepatica* contributes to completing a picture of the parasite as it migrates through the liver leaving extensive damage in its wake for the host to repair. The major players in the mechanism of tissue migration are familiar and well-characterised molecules such as cathepsin-like peptidases, leucine aminopeptidases, protease inhibitors, anti-oxidants and immunomodulators, which have been our prime candidates for vaccine development. The inconsistency in the performance of these vaccines [3, 79, 80], however, suggests that it will not be an easy task to stop the parasite in it tracks as its rapid physical growth in the liver renders the humoral and cellular components of the host immune system ineffective. It is therefore imperative that we further our understanding of how the host responds to early infection prior to the parasite entering the liver and also decipher the parasite mechanism(s) of immune manipulation if we are to conceive novel control strategies, particularly vaccines. Furthermore, a deeper scrutiny of how flukes may respond to environmental cues from the host in order to control their development and sexual maturity will open new avenues for novel anti-fluke drug design. The challenge now is to elucidate the signalling mechanisms that regulate the changes elicited by these cues. Since the completion of the first draft *F. hepatica* genomes [9, 81] we have been very successful in gathering fundamental developmental –omics data on the major intra-mammalian liver fluke stages, including this study, which will provide a rich source of information for future creative basic and applied endeavours.

## Methods

### Isolation of 21-day old immature flukes

Twenty-six BALB/c mice (male; 8 week old; Charles River) were infected orally with 30 *F. hepatica* metacercariae (Italian isolate; Ridgeway Research Ltd). At 21 days post-infection (dpi) the mice were euthanised by depletion of O_2_ using CO_2_, and the parasites recovered from the liver for total enumeration. A range of 1–12 parasites were recovered from 25 mice (no parasites were recovered from one mouse), resulting in 153 parasites, termed hereon in as 21-day old immature flukes. Fifty-seven immature flukes separated into three biological replicates of 19 parasites per group were washed in PBS and stored at − 80 °C prior to RNA extraction. The remaining 96 immature flukes, separated into three biological replicates of 32 per group, were washed and incubated for 24 h in pre-warmed (37 °C) culture medium (RPMI 1640 medium; ThermoFisher Scientific) containing 2 mM L-glutamine, 30 mM HEPES, 0.1% (w/v) glucose, and 2.5 μg/ml gentamycin). After the incubation, the parasites were centrifuged at 400 x g and the supernatant, the excretory-secretory (ES) proteins recovered for proteomic analyses. The parasites were washed in PBS and stored at − 80 °C prior to somatic protein extraction.

### RNASeq analysis & bioinformatics analysis

Total RNA was extracted using the miRNeasy mini kit (Qiagen) according to the manufacturer’s instructions, in a final elution of 50 μl RNase-free water. RNA integrity and concentration were confirmed using the 260/280 LVis plate functionality of the PolarStar Omega Spectrophotometer (BMG LabTech) and the Quant-iT RiboGreen RNA Assay Kit (ThermoFisher Scientific). Ilumina TruSeq RNA libraries (stranded) were prepared from three biological replicates of 21-day old immature parasites by Eurofins Genomics (Eurofins GATC Biotech GmbH) and sequenced (single read; 50 bp) on a HiSeq 4000 (Illumina), resulting in at least 38 million reads per sample. Illumina HiSeq reads were aligned to the gene models derived from the *F. hepatica* genome [9; PRJEB6687] using Bowtie (v2.2.9). Differential expression levels were determined as fragment per kilobase per million reads (FPKM) using Cuffdiff. Scatter plots and volcano plots were used to inspect the overall quality of RNASeq data and to assess the level of variation between the biological replicates, visualized using the cummeRbund package (v2.0.0; Additional file 1).

Transcript annotation was based on the gene model annotation carried by Cwiklinski et al. [9] using Uniprot, Gene Ontology (GO) and Interpro in silico tools and the KEGG Automatic Annotation Server (KAAS [82];) and manual curation of the cathepsin peptidases following the analysis reported by Cwiklinski et al. [36]. Metabolic pathway analysis was carried out by normalizing the global patterns of expression at the KEGG module level [10]. Semantic similarity analysis of GO terms was carried out using the web-based program, REVIGO [83] and graph-based visualization generated using the plotting R script generated by REVIGO.

Comparative analysis with the *F. gigantica* immature flukes recovered at 42- and 70-days post infection was carried using the previously published transcriptome dataset [20] based on GO analysis and the most highly transcribed genes calculated as FPKM. Enrichment of GO terms within the *F. hepatica* and *F. gigantica* datasets was determined using hypergeometric tests in R.

Investigations of oxidative stress associated with fasciolosis were carried out by interrogation of the mouse microarray data generated by Rojas-Caraballo and colleagues [11] using the gene fold change data presented within their Supplemental data file.

### Mass spectrometry analyses of somatic proteome and secretome

Somatic proteins were extracted from three biological replicates of 21-day old immature *F. hepatica* by homogenization in RIPA buffer (ThermoFisher Scientific) containing protease inhibitors (cOmplete Ultra tablets; Sigma Aldrich) and placed on ice for 30 min. The extracted proteins were centrifuged at 13, 000 x g for 10 min to remove any insoluble components, quantified using the Bradford assay (BioRad) and the supernatant stored at − 20 °C until use. The secreted proteins recovered from parasite culture of the 21-day old immature flukes were concentrated 6-fold using an Amicon Ultra 3 kDa column (Sigma Aldrich). For comparative analysis the secreted proteins were recovered from parasite culture of the newly excysted juveniles (NEJ; 24 h culture) and the adult liver fluke parasites (5 h culture) using methods as previously described [10, 84]. In each case the parasite isolate used was the Italian isolate (Ridgeway Research, UK). The adult secreted proteins were concentrated 30-fold using an Amicon Ultra 3 kDa column (Sigma Aldrich).

Protein digestion and mass spectrometry analyses were performed by the Proteomics Platform of the CHU de Québec Research Center (Quebec, Qc, Canada). Protein samples were acetone precipitated and subsequently solubilized in 50 mM ammonium bicarbonate and 1% sodium deoxycholate followed by reduction with 0.2 mM DTT at 37 °C for 30 min and alkylated with 0.9 mM iodoacetamide at 37 °C for 20 min. Trypsin digestion of the protein samples was performed in solution using 0.2 μg sequencing grade trypsin (Promega) overnight at 37 °C. The digested peptides were purified by stage tip (C18), vacuum centrifuge dried and then re-suspended in 0.1% formic acid, with final amount of 1 μg of protein being used for analysis by mass spectrometry. The re-suspended peptide samples were separated by online reversed-phase (RP) nanoscale capillary liquid chromatography (nanoLC) using a Dionex UltiMate 3000 nanoRSLC chromatography system (Thermo Fisher Scientific / Dionex Softron GmbH, Germering, Germany). Electrospray mass spectrometry (ESI MS/MS) analysis was performed on a Orbitrap Fusion mass spectrometer (Thermo Fisher Scientific, San Jose, CA,USA) utilising the Orbitrap Fusion Tune Application 2.0 and fitted with a nanoelectrospray ion source.

MS/MS peak lists (MGF files) were generated using Thermo Proteome Discoverer software (Thermo Fisher Scientific Inc., version 2.2.0) and analysed using Mascot (Matrix Science, London, UK; version 2.5.1), set up to search against a database comprised of gene models identified from the *F. hepatica* draft genome as utilised for the transcriptome analysis above (version 1.0, 101,780 entries; PRJEB6687 [9]), assuming digestion with trypsin with two missed cleavages permitted. Fragment and parent ion mass tolerance were set at 0.60 Da and 10.0 PPM, respectively. Carbamidomethyl of cysteine was set as a fixed modification and the deamidation of asparagine and glutamine, and oxidation of methionine specified as variable modifications.

Scaffold (version 4.8.4, Proteome Software Inc., Portland, OR) was used to validate MS/MS based peptide and protein identifications and calculate protein abundance using the Exponentially Modified Protein Abundance Index (emPAI). Peptide identifications were accepted if they could be established at greater than 95% probability to achieve an FDR less than 1.0% by the Scaffold Local FDR algorithm. Protein identifications were accepted if they could be established at greater than 95.0% probability to achieve an FDR less than 1.0% and contained at least 2 identified peptides. Protein probabilities were assigned by the Protein Prophet algorithm [85]. Proteins that contained similar peptides and could not be differentiated based on MS/MS analysis alone were grouped to satisfy the principles of parsimony. Putative annotation of the *F. hepatica* gene models was assigned based on annotation carried by Cwiklinski et al. [9] using Uniprot, Gene Ontology (GO) and Interpro in silico tools and the KEGG Automatic Annotation Server (KAAS [82];).

## Supplementary Information

**Additional file 1: Figure S1.** Quality assessment of RNASeq data generated from three biological replicates of *F. hepatica* 21-day old immature flukes. (A-C) Scatter plot comparison between the three datasets (Juv_1, Juv_2, Juv_3) generated using the cummeRbund package to inspect overall quality of RNA-Seq data. (D-F) Volcano plot comparisons between the three datasets (Juv_1, Juv_2, Juv_3) generated using the cummeRbund package to assess the variation between the three biological replicates.

**Additional file 2: Table S1.** Gene transcription by *F. hepatica* 21-day old immature flukes, represented as Fragments per Kilobase of transcript per Million (FPKM).

**Additional file 3: Figure S2.** Schematic representation of the significantly enriched gene ontology (GO) terms relating to biological processes within the *F. hepatica* 21-day old immature fluke transcriptome. The FDR adjusted *p* value is shown on the y axis. The bubble colour represents the transcription value of the genes associated with the GO term based on a log scale of the FPKM value. The circle size represents the number of genes associated with the GO term on the x axis. Description of the significantly enriched GO terms is presented in Additional file 4.

**Additional file 4: Table S2.** Enrichment of key gene ontology terms within the *F. hepatica* and *F. gigantica* immature liver-stage parasite transcriptomes.

**Additional file 5: Table S3.** Identification of proteins within the somatic proteome of *F. hepatica* 21-day old immature flukes by LC-MS/MS.

**Additional file 6: Table S4.** Identification of proteins within the secretome of *F. hepatica* 21-day old immature flukes compared with the secretome of NEJ 24 h and adult flukes by LC-MS/MS.

**Additional file 7: Table S5.** Protein abundance of proteinase inhibitors within the *F. hepatica* life cycle stage secretomes.

**Additional file 8: Table S6.** Differential gene expression of genes associated with fibrosis and inflammation, oxidative stress, and proline metabolism within liver tissue of mice infected with *F. hepatica*. Fold change analysis based on expression of non-infected animals versus mice infected for 21 days, based on data from Rojas-Caraballo et al. [11].

## Data Availability

The transcriptome data sets supporting the conclusions of this article are available in the Sequence Read Archive (SRA), under the accession PRJNA665699; https://www.ncbi.nlm.nih.gov/sra/PRJNA665699. The mass spectrometry proteomics data have been deposited to the ProteomeXchange Consortium via the PRIDE partner repository with the dataset identifier PXD021221 and 10.6019/PXD021221.

## Abbreviations

ES: Excreted Secreted proteins
FABP: Fatty Acid Binding Protein
FDR: False Discovery Rate
FPKM: Fragments per Kilobase of transcript per Million
GO: Gene Ontology
GPx: Glutathione Peroxidase
GRX: Glutaredoxin
GSS: Glutathione Synthetase
GST: Glutathione S Transferases
HDM: Helminth defence molecule
NEJ: Newly Excysted Juveniles
PRX: Peroxiredoxin
ROS: Reactive Oxygen Species
SOD: Superoxide dismutase
TLR: Toll Like Receptor
TGR: Thioredoxin Glutathione reductase
TRX: Thioredoxin
WHO: World Health Organisation
