# Supplementary figures and images for: Complementary transcriptomic and proteomic analyses reveal the cellular and molecular processes that drive growth and development of Fasciola hepatica in the host liver
Source: BMC Genomics. 2021 Jan 11;22:46. doi: 10.1186/s12864-020-07326-y (PMC7797711; doi:10.1186/s12864-020-07326-y)

## Slide 1
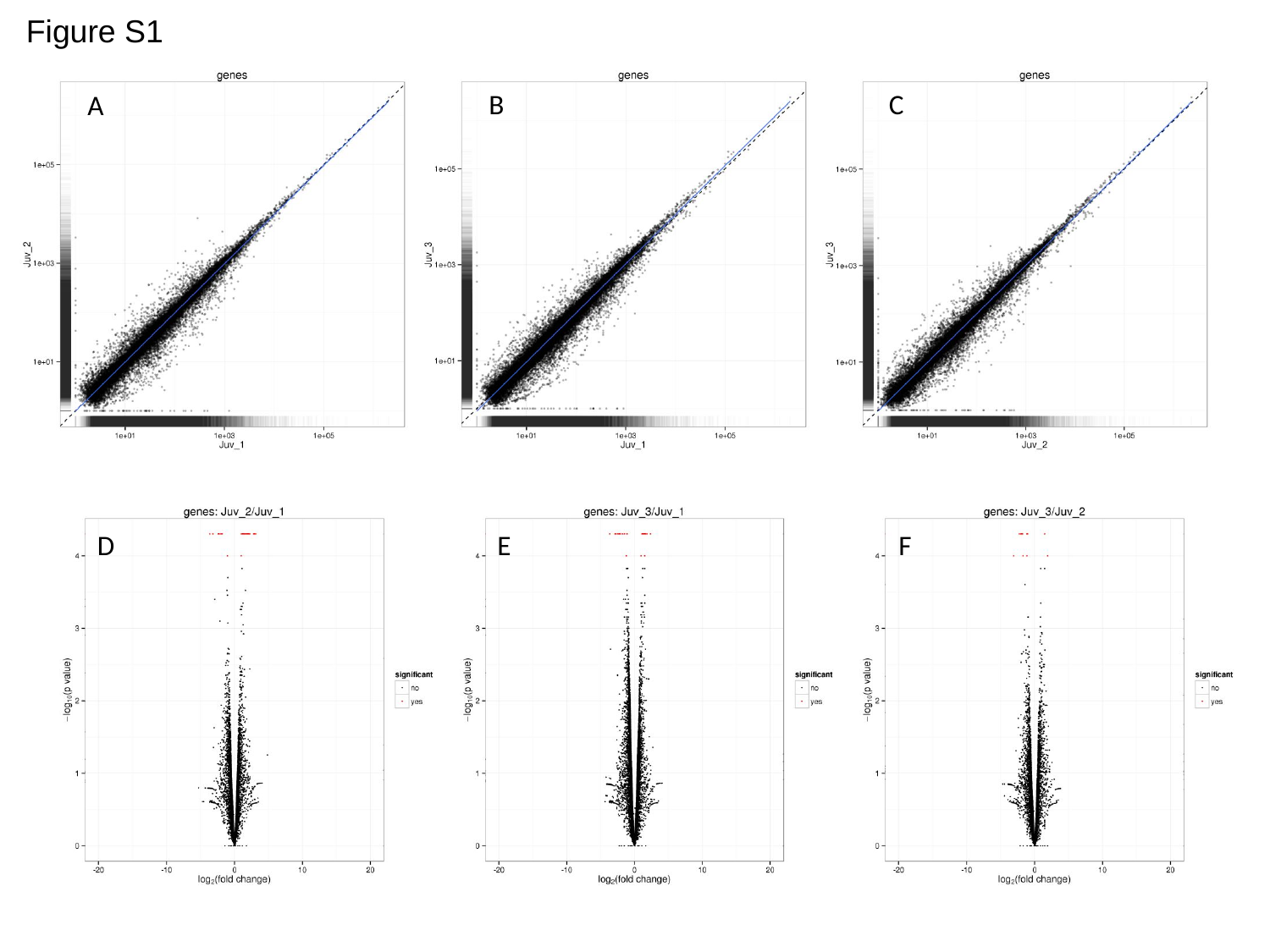

Figure S1
B
C
A
D
E
F

Supplement: Supplementary file 1 — Additional file 1: Figure S1. Quality assessment of RNASeq data generated from three biological replicates of F. hepatica 21-day old immature flukes. (A-C) Scatter plot comparison between the three datasets (Juv_1, Juv_2, Juv_3) generated using the cummeRbund package to inspect overall quality of RNA-Seq data. (D-F) Volcano plot comparisons between the three datasets (Juv_1, Juv_2, Juv_3) generated using the cummeRbund package to assess the variation between the three biological replicates. [file 12864_2020_7326_MOESM1_ESM.pptx]

## Slide 1
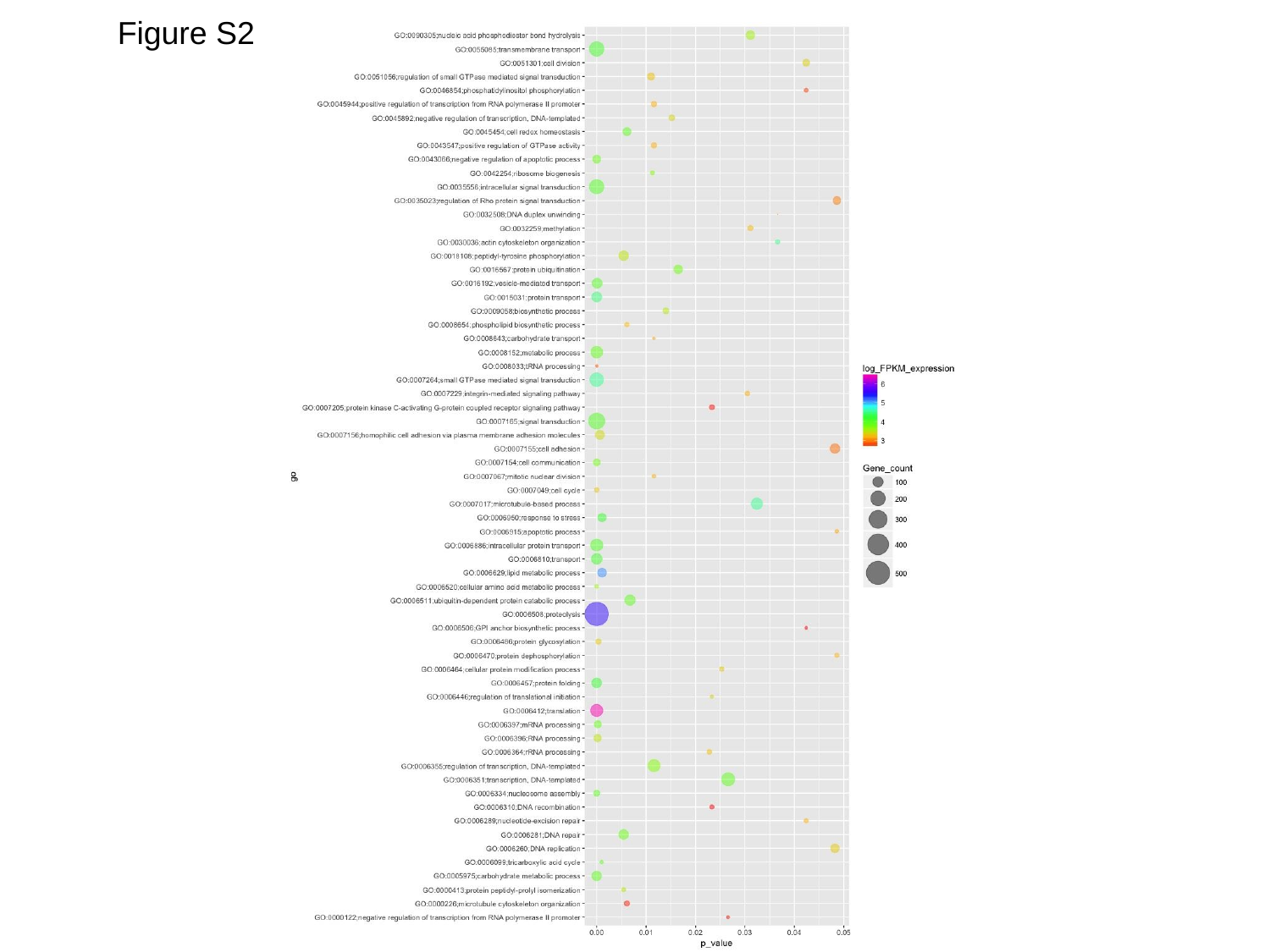

Figure S2

Supplement: Supplementary file 3 — Additional file 3: Figure S2. Schematic representation of the significantly enriched gene ontology (GO) terms relating to biological processes within the F. hepatica 21-day old immature fluke transcriptome. The FDR adjusted p value is shown on the y axis. The bubble colour represents the transcription value of the genes associated with the GO term based on a log scale of the FPKM value. The circle size represents the number of genes associated with the GO term on the x axis. Description of the significantly enriched GO terms is presented in Additional file 4. [file 12864_2020_7326_MOESM3_ESM.pptx]
