# Supplementary material for: Complementary transcriptomic and proteomic analyses reveal the cellular and molecular processes that drive growth and development of Fasciola hepatica in the host liver
Source: BMC Genomics. 2021 Jan 11;22:46. doi: 10.1186/s12864-020-07326-y (PMC7797711; doi:10.1186/s12864-020-07326-y)
Supplement: Supplementary file 7 — Additional file 7: Table S5. Protein abundance of proteinase inhibitors within the F. hepatica life cycle stage secretomes. [file 12864_2020_7326_MOESM7_ESM.docx]

**Additional File 7: Table S5.** **Protein abundance of proteinase inhibitors within the *F. hepatica* life cycle stage secretomes.**

|  | Stefins/Cystatins | Serpins | Kunitz-type |
| --- | --- | --- | --- |
| Immature flukes | 8.68 | 2.57 | 282.79 |
| NEJ | 18.63 | 8.61 | 4.88 |
| Adult flukes | 35.45 | 7.08 | 5.94 |
